# Supplementary material for: Decreased Spikelets 4 Encoding a Novel Tetratricopeptide Repeat Domain-Containing Protein Is Involved in DNA Repair and Spikelet Number Determination in Rice
Source: Genes (Basel). 2019 Mar 13;10(3):214. doi: 10.3390/genes10030214 (PMC6471630; doi:10.3390/genes10030214)
Supplement: Supplementary file 1 [file genes-10-00214-s001.zip › Supplemental data 1.docx]

**>Genomic sequence of *DES4***

ATGGGGCGCGGCGGCGGGGGACGGTGGACGAAGGAGGAGGAGGAGGAGCTGAAGGCGGCGAAGCGGGGGTACCGGGAGGCGGTGGCGGAGGGGAACCGGGAGGAGGAGGCGCGGTGGGCGAACGTCATCGGCGACATCCACAAGCGGCGGGGGGAGTACGTGGAGGCGCTCCGGTGGCTGCGGATCGACTACAAGGTCTCCGTCAAGTACCTCCCCCAGCGCCACCTCCTCCCCTCCTGCCAGTCGCTCGGCGAGGTCCACCTCCGCCTCGGCAACTTCTCCGAGGCCCTCACCTACCAGAAGAAGCACTTACAACTTGCCAAGGAGGCTGATGACCTTGTTGAGCAGCAGAGAGCTAGCACCCAGCTTGGCAGAACTTACTATGAGATCCTTCTAAGATCTGAAAATGATCACAGTGCCATTCGGAATGCCAAGAAATATTTCAAATCATCCATGAAGCTAGCAAGGGTTCTAAAGGAGAATCCATCGTCTCAGAAGTCTCTCTTCCTAAAGGAGCTTATTGATGCGTACAATAATATGGGCATGCTTGAACTGGAACTGGATAATTATGAAGAAGCTGAGAAATTACTTGTTCAGGGTCTGAAGATATGTGAGGAGGAAGAGGTACACCAGTATGACGATGCTCGCAGTAGGCTCCATCACAATCTAGGTAATGTTTATATTGAACTACGCAACTGGAATAGAGCCAAGGGCCATATTGAGAAGGACATAGAGATATGTAGAAAAATACGCCATACTCAAGGCGAGGCAAAGGGATTTATAAATCTGGGGGAGGTGCATTCCCGTGTTCAAAAGTATGAAGATGCAAAGCTTTGTTACAATAAAGCTCTTCAAATAACAAAGTGCTTGGAAGATGAGGATGCACTAATGGACCAAATCCATCAGAATATTGAAACTGTTACCAAAGCAGCCAAAGTACTTGAGGAAATGAAGACAGACGAGCAGAAACTGAAAAAGCTTGTCAGAGACACATCTAATGCTCGTGGAACATCTAAAGAGAGGAAGCTCCTCCTTGAGCAGTATGCTTGGCTGGATAATCTTATGGAGAAGGCAAGGATGATAACTGCATGGCCAAAACTGAAGGAATTTTCGAAAGGACAAAAGAGGGTAGCAAATGAACTCCATGACAAGGAAAAACAGAGCAACTCTCTTTTGGTCATAGGAGAATCTTACCAAAAGCTTAGAAACTTCAGCAAAGCTCGTAAATGGTGCATGAAAAGCTGGAATATGTATCGATCGATCGGAAACTTGGAGGGTCAAGCATTAGCAAAAGTGAACATTGGGAATGTTCTTGATTCTTGTGGTGACTGGGCTGGTGCGTTGCAAGCTTATGAAGAAGCATACAGAATTTCTGTGGAAGGTGGTCTTTCAAATGTGCAACTAGATGCCCTTGAAAACATGCATTACAGTCACATGGTCAGATTTGATAACATTGAAGAAGCGAAGAAACTGCAGCAAGAAATAGACAGCCTGAAGAGAATGTCTGATCAACATGAAGCAAGGGATACAGTTAGTGACTATTGCTCAGAAACTGAAAGTGAAGATGGCAATGTATCTGATAATATCCTCAATACAGAGGACAATGATGGGAATATTGCAAATAATATTTCTGAGGAATTTGATGATGATGTTGTTCTTGCCTCACTTGTTCATAAAAGTAAGTCATCTAAGACCAAAGCATCTAAGATACATAGTAGTCCGAAGAATGTTGATGAGTCATGTGACATGGATGGGAGCCCTGAAGAAGTTGTAAGTAAATCATTTAGCAACCATTCTGGTAGAAAGCGTGTTCGAGTTGTCATATCAGATGATGAAGCTGAAGAAGCTCCTGAAATTGATCAGTCAAAAAGAACACTTACTGGTCGAGCAGATAGTCTGTCTACCTCAGAGCGAATTGCAAATGCAGCAAACAGAAACAGAAATCAGCATACCTCTCATCCCATTGAGACCAAAGAAGTAGACAGTGTTTGCACTCCCTGCCCTGCTGAGGAAAGTATATGTTCATTCAAATCTGGTAGTCCTGTTTGTCATGGTAATGATGGCCCAGATTTAGGAGCTTCTAGCATAGGAAAGTTATCTGTGTCCAAACCAGCAGCGAGTGGTTCCAAGGTTGGTACACATGCATCAAACAGTCGGCCCCAATGTCAAAATGCTGTTGGTCTTCAATCCTCAGATGCTGATCATAAGTTCTGGGTGTTCAAAATTGGTGAACTTTTGGTTTACTTGGATGCAAATGCATGTACATGTGAGGGTGCCTTTAGCATTGAGTGTCTTAAAGTTGAAGTGGCATGCGTGTACTATCTTCAGATTCCTGATGAGAAGAGGTCCAAAGGTTTGTTGCCTATTATTGGGGAACTCAAGTGTTGTGGGAAGGTACTAGATGACACGGACTCACGAGATTATATTGATCAACTTGCCTCTGAACAGAAGTGCATTGATGTTGTTATTGATGATTGGGTGCCAAAGCGGCTGATGAAATTGTATGTTGATTTCTGCACAAAGTTATCAGAAGCGCCAAATAAGAAGCTCTTAAAGAAATTATATAATCTTGAAGTCTCCGAAGACGAGGTTATTGTGTCTGATTGTGGACTCCAAGACCTGTCGATCACACCTTTTCTTGATGCTCTAAGATTACATAAAACAATAGCTGTGTTGGATCTTTCCCATAATATGCTAGGAAACCAAACAATTGAGAGGCTTCAACAAATATTTTCTTCATCAAGCCAAACATATGGTGGCTTGACACTGGATTTACATTGTAATCGATTTGGTCCAACTGCTTTATTTCAGATATGTGAGTGCGCTGTTATGACTAATCGATTGGAAGTACTTAATCTGTCTGGGAATCGCCTCACAGATGCATGCGGTTCTTACCTTTTCACAATCCTACAGAAGTGCAAAGCACTGTACAGCTTGAATGTCGAGCAATGTTCTATCACATCAAGAACAGTTCAGAAGATGGCAGATGCACTGCATGAAGGGTCTGCCCTTTCACATCTCTCCTTAGGAAACAACAATCCAATTTCTGGAAATACAATGCTTAGCCTTCTCTCCAAACTTGCCTCTCTGAAAAGGTTTTCAGAACTAAGCCTGACTGGTATAAAACTGAGTAAGTTAATGGTTGATAAGCTGTGTGTACTTGCACAATCCTCATGCTTATCAGGATTTCTGCTAGGTGGAACTTACATTGGATCAGGAGGGGCAACTAAGCTTACTGAGGCACTCTCTTGTGCATCACAAGAATTGCTGAGATTGGATTTATCAAACTGTGGGCTTACAACTCCTGATTTCTCGCAACTCTGTACAAATCTTTCTCAAATTAATATCGTTGACTTGAACCTTGGAGGCAATTCTTTTACCCTAGAGGAATGTGATGCTATTAGGGCATTGCTTTCGAATCCCCAATGCAGTCTCAGATCTCTCACCCTTGATAGATGCAATCTTGGGCTTGCTGGCACCGTGGGCATTATTCAAGCACTAGCAGGAAATGACCAATTGGAGGAACTACGCGTTGCTGAGAACACAAACCTGGCACTACAGAGAACATTGCAGTACGATGAAGACGCGCAGGATGTATCACCAGGTACTGACCAAAACCAACGTACCAATGCTGAAGCGAACGATCACATAGACCCTGATAAGATGGAGGTGCCGGATAGCGAAGACGAAGAGGCAGTCCACGAGGACACCCGTGCAGCGACCGGCCCAGACGGGAGCTGTGCAAGTTCATGCCAGAGAAACTCCTCTTCTGGCTGTCATGCCATCCAAGAACTCGCTGATGCCATCATTTCTGCAAAGCAACTGAAGGTGCTCGATCTCAGTCGGAACGGGTTGTCGGAAGAGGATATCCAGTCACTGTATTCTGCTTGGGCTTCTGGCCCTAGAGGTGATGGAATGGCTCGGAAACATGTAGCCAAGGAGGTGGTGCATTTTGCAGTGGATGGGATGAATTGCTGCGGCCTGAAGCCCTGCTGCAGAAGAGACTTGCAGATGTAG

**The highlighted C was deleted in *des4*.**

**>protein sequence of DES4**

Arrow indicates the frame shift and premature of mutated protein.
