# Supplementary material for: Decreased Spikelets 4 Encoding a Novel Tetratricopeptide Repeat Domain-Containing Protein Is Involved in DNA Repair and Spikelet Number Determination in Rice
Source: Genes (Basel). 2019 Mar 13;10(3):214. doi: 10.3390/genes10030214 (PMC6471630; doi:10.3390/genes10030214)
Supplement: Supplementary file 1 [file genes-10-00214-s001.zip › Supplemental figures.docx]

**
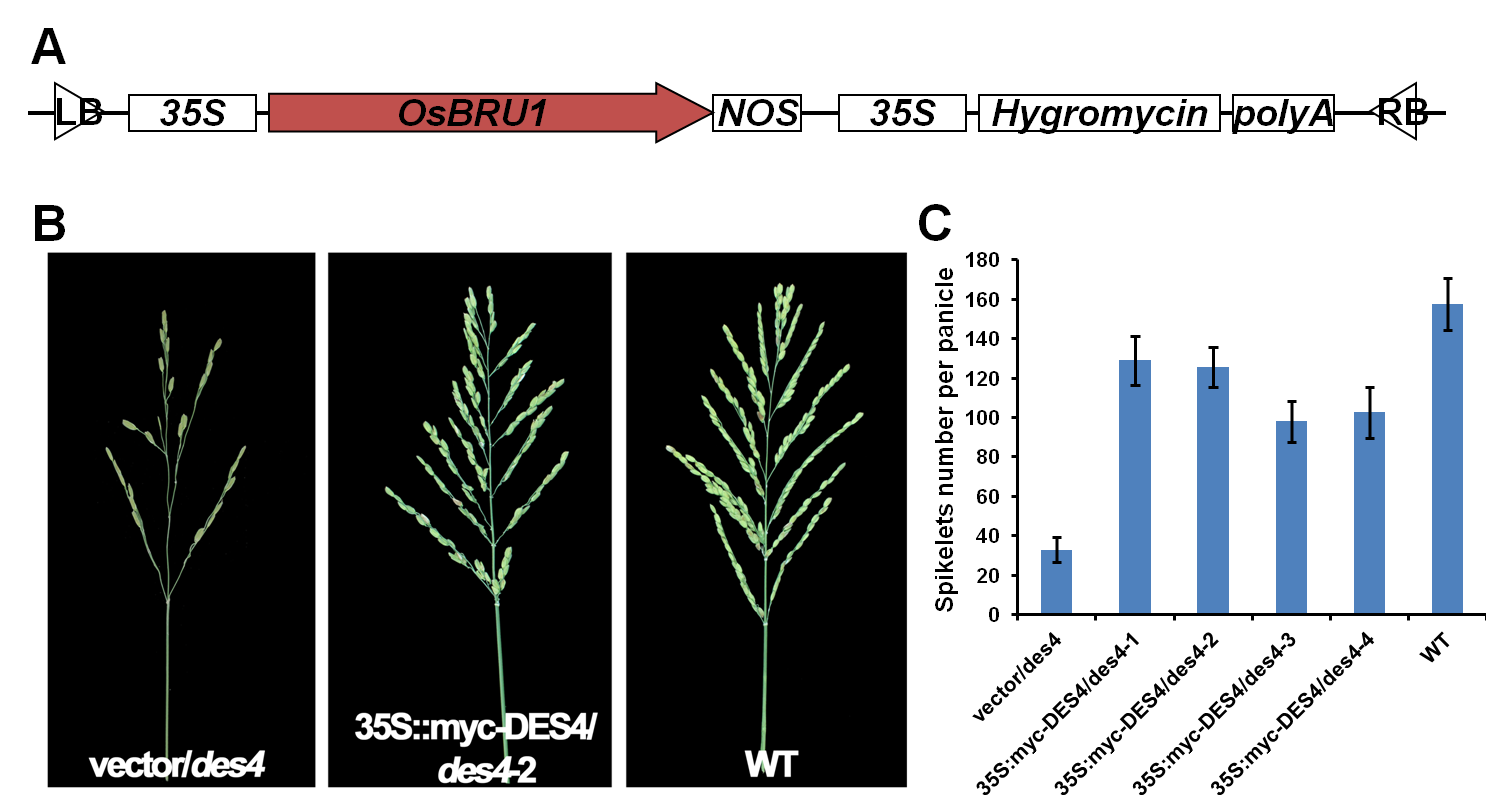
Figure S1.** The spikelet numbers of genetically complemented *des4* lines.

**Figure S2**. qRT-PCR analysis of *DES4* in various rice tissues.

R:root; AB: axillary bud; In: internode; N: node; LB: leaf blade; P: young panicle; SP: spikelet. Values are means ± SE. Primers could be found on Table S1.


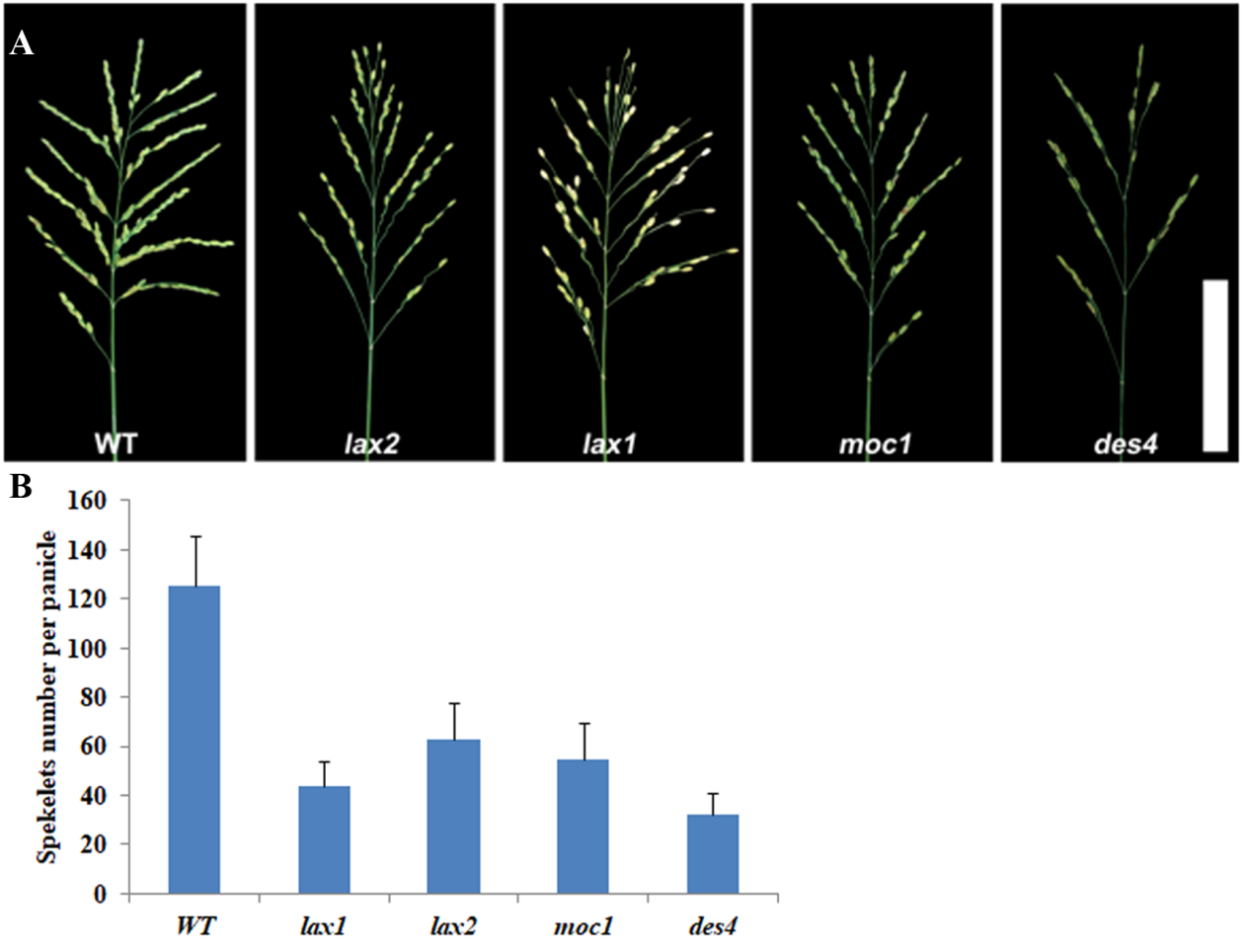


**Figure S3.** Panicle morphologies of *lax1, lax2, moc1* and *des4*.

A. Typical panicle morphologies of the four mutants. Bar=15 cm; B. quantification of spikelet numbers per panicle of the four mutants. Data is presented as mean ± SD (n=15).
